# Importance of rotary systems in dental care by undergraduate students in patients of a public health service of Belo Horizonte

**DOI:** 10.4317/jced.52663

**Published:** 2016-02-01

**Authors:** Fernanda-Alcaraz-Orta Bruno, Eduardo Nunes, Martinho-Campolina-Rebello Horta, Ana-Maria-Abras da Fonseca, Frank-Ferreira Silveira

**Affiliations:** 1 DDS, MS, Graduate student, Departament of Dentistry, Pontifícia Universidade Católica de Minas Gerais, Belo Horizonte, Brasil; 2 DDS, MS, PhD, Professor, Departament of Dentistry, Pontifícia Universidade Católica de Minas Gerais, Belo Horizonte, Brasil; 3 DDS, MS, Professor, Departament of Dentistry, Pontifícia Universidade Católica de Minas Gerais, Belo Horizonte, Brasil

## Abstract

**Background:**

The aim of this study was to evaluate the fracture rates of ProTaper rotary files used in the endodontics extension clinic of the Undergraduate Dentistry Course of the Pontifical Catholic University of Minas Gerais during the prior eight years.

**Material and Methods:**

Control record data regarding file usage by students were collected and analyzed by semester. For each period, the total number of patient consultations, the frequency of file use, the occurrence of fractures and the file numbers for which fractures occurred were noted. Descriptive statistics, including frequency of file fractures were calculated for all file types. The chi-square test was used to evaluate differences in the frequency of file fractures between all file types.

**Results:**

The study results revealed that during the examined period, there were 1006 consultations and 7993 uses of files. A total of 30 file fractures were recorded throughout this period; thus, fractures occurred in 0.37% of total file uses and 2.98% of all consultations. The most frequently used files were S1, S2 and F1, and these files also accounted for the most fractures. However, no differences in the frequency of file fractures were observed between the file types (*p*𰀎0.05).

**Conclusions:**

The low fracture rates observed in this study indicate that the examined instruments can be used to safely provide dental care to patients.

** Key words:**Dental instruments, endodontics, public health.

## Introduction

The effective cleaning and shaping of the root canal system is the most important factor affecting the success of endodontic therapy (1). Cleaning and shaping procedures seek to remove organic and inorganic material from within the root canal to reduce the number of microorganisms in the canal, neutralize endotoxins within the dentin, and prepare the root canal for proper closure (2).

The preparation of straight root canals is relatively uncomplicated; however, even for seasoned professionals, atresic root canals or root canals with severe curvatures present difficulties related to the potential for iatrogenic defects. The characteristic stiffness of stainless steel files hinders the use of these files in the cleaning and preparation of root canals with severe curvatures (3). Despite the introduction of flexible nickel-titanium (NiTi) instruments, the preparation of severely curved root canals remains challenging (4).

The development and utilization of NiTi files has led to great advances in endodontic treatment; relative to stainless steel instruments, NiTi instruments can produce not only higher treatment quality and greater treatment speed but also fewer failures (5). NiTi instruments are sturdy, have good biocompatibility, and exhibit the notable characteristic of superelasticity, which refers to the ability to recover to an initial shape simply through the removal of stress, with no heating required (6,7).

In clinical practice, the main disadvantage of NiTi instruments is the possibility of unexpected instrument fracture within the root canal, an event that compromises treatment success. These fractures can occur due to either torsion or flexural fatigue (8). When an instrument is held within the root canal, static torsion occurs if one end of the instrument twists while the other end continues the rotary movement of the motor. Dynamic torsional fracture results from the friction produced by the shear strength of dentin in response to a file.

In the context of endodontic treatment, during automated instrumentation in curved canals, rotation causes files to be subjected to tension and compression forces concentrated on the point of maximum curvature of the instrument within the root canal (1,9). These forces can lead to instrument fracture due to flexural fatigue; in particular, the instrument may be subjected to cyclic deformation that places stress on the alloy, leading to the nucleation of cracks that ultimately expand to produce fractures. It is therefore important to assess the internal geometry of curved root canals prior to treatment. Relevant measurements that are generally obtained include a canal’s angle and radius of curvature. These parameters are extremely important for evaluating root canal instrumentation (10,11).

The possibility of intracanal instrument fracture is the underlying motivation for studies that attempt to identify factors that may increase the risk of instrument failure and establish parameters for the safe and efficient use of NiTi files in automated systems. These approaches reflect the fact that the best treatment for intracanal file fracture is to prevent such fractures from occurring.

During endodontic treatment using automated NiTi file systems, the risk of intracanal file fracture is affected by multiple factors, such as the anatomy of the root canal (12), the angle of curvature of the canal, rotation speeds and torque (10,12), and how frequently files are reused (13).

The increasingly frequent use of rotary systems in endodontic treatment underscores the need to provide students with adequate clinical training with these systems. The present study sought to evaluate the fracture rates of the ProTaper rotary files used by undergraduate dental students over an eight-year period.

## Material and Methods

This study was approved by the Pontifical Catholic University of Minas Gerais (PUC Minas) Research Ethics Committee (proto-col number CAAE – 10732812.1.0000.5137). The present study was based on data regarding treatments performed using the ProTaper system (Maillefer, Baillaguess, Switzerland) during the prior eight years in the endodontics extension clinic of the Undergraduate Dentistry Course PUC Minas. In particular, data collected in the file usage control records, organized by student and semester, were utilized in this investigation. The endodontics clinical extension is an optional component of the dentistry undergraduate program at PUC Minas; during this extension, students are supervised by teachers who have experience with automated endodontics. The protocol adopted for this study advocates the disposal of instruments after five uses.

For each semester of the examined period, data relating to total consultations, the number of times each ProTaper file was used, the occurrence of fractures, and which files in the recommended sequence fractured were collected and assessed.

A total fracture index relative to total consultations and the number of uses of files was established. In addition, this fracture index was stratified by file type in the file sequence advocated in the protocol adopted in the examined clinical extension.

Descriptive statistics, including frequency of file fractures were calculated for all file types. The chi-square test was used to evaluate differences in the frequency of file fractures between all file types. The level of significance was set at 5%. The analyses were performed using GraphPad Prism Software (GraphPad Software, San Diego, California, USA).

## Results

No differences in the frequency of file fractures were observed between the file types (*p*>0.05). The data obtained in this study are presented in  Table 1. These data indicate that during the examined period, there were 1006 consultations and 7993 uses of files. A total of 30 file fractures were recorded throughout this period; thus, fractures occurred in 0.37% of total file uses and 2.98% of all consultations.

**Table 1:**
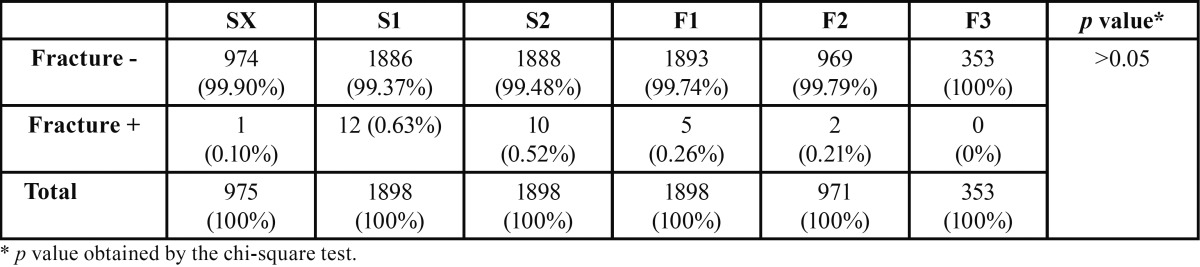
Comparison of file fractures between all file types.

## Discussion

The appropriate cleaning and preparation of the root canal system is an essential step in successful endodontic treatment. Rotary NiTi files are currently being utilized in endodontic practice; relative to stainless steel files, NiTi files provide quality, efficiency, comfort, and safety advantages for both dentists and patients. However, the occurrence of unexpected instrument fracture within the root canal is a commonly reported problem. This issue underscores the importance of not only studies addressing the use of rotary systems but also the training of professionals and undergraduate students.

Prior research has indicated that the technique and skill of the operator of an automated system are correlated with the incidence of unexpected intracanal instrument fracture (7). However, a study has revealed that inexperienced dental students can use rotary systems to adequately prepare curved canals while preserving tooth structure; in fact, these students committed few procedural errors and completed procedures more quickly when using rotary systems than when using hand instruments (14). These findings have been corroborated by researchers who observed the occurrences of fractures and instrumentation times during the preparation of curved molar canals by students without clinical endodontic experience and concluded that the ProTaper rotary system can be utilized appropriately by students without clinical experience if these students have received basic training with the equipment (15).

A study that evaluated students’ perceptions regarding endodontic dental treatment with rotary NiTi instruments and with manual stainless steel instruments indicated that training in the use of automated systems should be included in the undergraduate dentistry curriculum to improve students’ levels of clinical experience (3). Another investigation has described cases in which training with NiTi rotary instruments was successfully introduced into an undergraduate program in endodontics (16).

A recent study that sought to identify factors affecting the fracture of ProTaper files reused in clinical treatment with a rotary system indicated that fractures occurred in 2.6% of treated teeth, which corresponded to 1.1% of the number of treated canals (17). Although these researchers concluded that instrument fracture is a multifactorial process, they found that the type of file that is most frequently fractured is F3 because of this file’s relatively large diameter; this finding corroborated concerns that F3 files are relatively susceptible to cyclic fatigue fracture (18). In contrast, consultations in the extension clinic of the current study prioritized the treatment of molars; thus, the F3 file was not the most frequently used type of file. This phenomenon may explain why a higher incidence of F3 fractures was not observed.

Given the number of consultations considered in this investigation and the low observed incidence of instrument fracture, the results of this study should encourage the academic community to review extremely well-established paradigms in endodontic practice.

The Department of Dentistry of PUC Minas seeks to provide undergraduate students with appropriate clinical training in rotary NiTi systems that use ProTaper files. In accordance with this objective, undergraduate students admitted to the endodontics extension clinic receive specific instructions and basic theoretical preclinical training; this preparation has proven to be adequate to allow these students to begin using rotary systems in endodontic treatment.

All treatments performed in the extension clinic are controlled by the completion of worksheets that indicate the identities of the student and the responsible teacher, the treatment period, the date of consultation, the patient’s record, the treated tooth, the number of uses of the ProTaper files, and the occurrence of instrument fracture.

Due to the significant number of patient consultations during the period examined in this study and the low number of file fractures during this period, a low overall incidence of instrument fractures (2.98%) was observed. An extremely important consideration is that each file was used five times (for five teeth); as a result, the small number of fractures was a relevant finding.

Regarding the root canal treatment performed in a conventional manner, the rotary system has a gain in productivity and quality, especially with teeth anatomical complexity. Therefore, professionals working in the public health service should be encouraged to use this technology, with significant reduction in the time taken for completion of the treatment, which can reverse in a greater number of people served.

## Conclusions

The results of this study demonstrate that ProTaper files exhibited a low fracture rate and indicate that the materials and practices used in the endodontics extension clinic of the Undergraduate Dentistry Course of PUC Minas are efficient for providing safe and effective care.
